# Analectic

**Published:** 1835

**Authors:** 


### MISCELLANEOUS INTELLIGENCE.

### ANALECTIC, ANALYTICAL, AND ORIGINAL.

### I. Analectic.

*Ad Sylvas, Nuncius.*

1. *Abstract of Observations on Motions and Sounds of the Heart.* By Hugh Carlile, A. B. Demonstrater in the School of Anatomy in the University of Dublin.—The circumstances in the history of the heart's action which have been most the subject of controversy within late years may be enumerated as follow:—1st, the expansion and contraction of the auricles and ventricles, commonly called their "systole" and a diastole;" 2d, the beat of the heart against the side of the chest; 3d, the arterial pulse; and 4th, the sounds perceptible during the heart's motion. With a view to the explanation of these phenomena, the author has made some experiments on living animals, the results of which he was desirous of communicating to the Association.

In experiments of this kind it is desirable, as well for ensuring the means of accurate observation as for the sake of humanity, to diminish as much as possible the suffering of the animal. This can be accomplished by the use of the artificial respiratory apparatus, the animal having been suddenly deprived of sensation without shedding its blood. But the author has found that the application of this apparatus causes the heart to continue and terminate its motions in an unusual manner, and is therefore liable on some points to mislead the observer. In those cases in which the employment of artificial respiration is not expedient, there is much advantage in using very young animals for experiment. In this stage of life, as well as in animals of the inferior classes, the different organs appear to have a comparatively independent existence; and as their functions are in many instances performed with little disturbance under serious injury to the individual, they also retain their vitality long after their separation from the rest of the system. From the same causes very young animals appear to suffer less pain during experiment than those of mature age.

After discussing the methods of experimenting, the author proceeds to describe the opinions which have been held by other persons on the subjects in question, and to compare them with the conclusions to which his experiments have led.

1st. It has been asserted by Bichat, and his celebrity has induced many to adopt the opinion, that the ventricles possess a power of active dilatation, by means of which, when their systole has terminated, they are enabled to invite into their cavities the blood from the neighboring auricles. The author, however, has ascertained by experiment, that there is no such dilating power in the ventricles, but that these muscles, when their state of contraction has ceased, become perfectly soft and flaccid, like all other muscles in their state of repose, and thus readily admit the blood from their respective auricles, which had become distended during the systole of the ventricles. The feeling of resistance which was mistaken by Bichat for a dilating power, and was supposed by him to accompany the diastole of the ventricles, the author has ascertained to be caused by the swelling of their muscular fibres during their systole.

The auricles contract but little upon their contents in man and in the higher classes of animals, the small quantity of blood which the ventricles discharge at each contraction being compensated by the frequency of their movement; while in the cold blooded animals, in which the heart acts with less frequency, the degree of expansion and contraction of both auricle and ventricle is much greater than in the former classes, and the quantity of blood sent through the heart at each movement is much larger.

2d. The impulse of the heart against the side of the chest, commonly called its beat, has been explained by different writers in various ways. Mr. Hunter supposed it to have been caused by the straightening of the curve of the aorta during the systole of the ventricles, whereby the apex of the heart was thrown forwards. Meckel refers it, in part, to the elongation of the arterial tubes during the ventricular contraction, and partly to the swollen state of the auricles at that time, by which the ventricles are pushed forward against the side of the chest. Harvey mentions an opinion held by some in his time, and which had been lately revived, namely, that the beat is caused, not by any active power in the ventricles, but by the muscular contraction of the auricles during their systole, by which the blood being sent with force into the ventricles, distends their cavities, and causes them to strike against the chest. This opinion, therefore, supposes the beat of the heart to coincide with the ventricular diastole. Various other suppositions have been put forward upon this subject by different authors.

The author's experiments show that the beat of the heart is coincident with the systole of the ventricles, and is caused by the peculiar shape which these parts acquire in their contracted and hardened state, their middle part becoming globular and prominent, and their apex being, as Hunter expressed it, "tilted" forward. During their systole the ventricles, like other muscles in a state of contraction, become swollen and hard to the touch, as was observed long since by Harvey. The greatest quantity of muscular fibre being situated about their middle part, where the "musculi papillares" are placed, this part during the systole assumes a globular and prominent form, projecting in front, and by its protuberance behind pushing forward the body of the ventricles. The apex is "*tilted*" forward for the following reason. The author has ascertained, by unravelling the structure of hearts prepared by boiling, that the fibres which pass from the base to the apex, on the front of the ventricles, are considerably longer than those similarly placed behind. In some human hearts he has found them in the ratio of five to three; the shape of the ventricles being nearly that of an oblique cone, whose base is applied to the auricles, and whose longest side is in front. Now, it is a law of muscular action, that fibres are shortened during their contraction in proportion to their length when relaxed. For instance, if a fibre one inch long, lose by contraction one-fourth of its length, or one quarter of an inch, a fibre two inches in length will lose *one* inch by a contraction of equal intensity. We have seen that the fibres, which, by their contraction cause the apex to approach the base of the ventricles, are much longer on the front than on the back part, and, consequently the former are more shortened during their contraction than the latter. The apex, then, does not approach the base in the line of the axis of the ventricles, but is drawn more to the side of the longer fibres, that is, towards the front, thus producing the "tilting" forward.

This conclusion is strengthened by the fact, that the forward motion of the apex of the ventricles is always proportioned to the obliquity of the form of these cavities in different classes of animals. In the heart of some reptiles, the frog for example, in which the lengths of the fibres of the ventricle before and behind are nearly equal, the tilting of the apex is scarcely discernible. The obliquity is greater, as far as the author has been able to observe, in the human heart than in that of any other animal.

Mr. Carlile has ascertained, also, that the ventricles assume this form during their contraction, after they have been separated from the auricles by a ligature, and even after they have been removed from the body, and placed in a vessel of tepid water, or held upon the hand, the auricles having been previously cut off; in all which cases the peculiar motions which accompany their contraction and relaxation were observed to recur as long as their power of moving remained; proving that the heat of the heart is produced altogether by the action of the ventricles during their systole, and that in these, as in all other muscles, the peculiar forms assumed during their contraction depend upon the relation, as to length and position, of the fibres of which they are composed.

3d. The arterial pulse, which is produced by the jet of blood sent from the left ventricle into the aorta during its systole, has been stated by Bichat and many other writers to be synchronous throughout the whole arterial system. But the experimenter can ascertain in his own person that the pulse is successive at different distances from the heart. If the hand be placed over the region of the heart, and the radial artery be felt at the same time, an interval will be distinctly perceptible between the beat and the pulse; and if the anterior tibial artery be substituted for the radial, the interval will be found still greater. Repeated observations of this kind show that the intervals of time between each beat of the heart and the corresponding pulse in different parts of the body are proportioned to the distances measured along the arteries, from the heart to the respective parts; and a knowledge of this fact leads, without further anatomical inquiry, to the conclusion that the beat of the heart is coincident with the ventricular systole. For, as the intervals of time between the beat and pulse are proportioned to this distances from the heart to those parts where the pulses are felt, it follows that when the distances become evanescent the intervals of time will also vanish. Consequently, at the origin of the aorta the pulse will coincide as to time with the beat of the heart; but the pulse at the origin of the aorta is necessarily synchronous with the ventricular systole, by which the blood is driven into that artery; and therefore the beat of the heart will coincide with the ventricular systole, a conclusion which agrees with that drawn from positive experiment.

The proportion which exists in the pulse between the intervals and distances is dependent upon the elasticity of the arteries.

4th. An explanation of the *sounds* of the heart has become necessary since the employment of the stethoscope in ascertaining the state of internal parts. Laennec has well described these sounds, and properly refers the first to the rush of blood from the ventricles during their systole. But, in supposing that the second sound is produced by the auricular systole, he has fallen into an extraordinary error, as the second sound follows immediately after the first one, whereas the auricular systole precedes the ventricular. This mistake has been noticed by different writers since Laennec's time, who have rejected his explanation, and substituted others in its place.

From the observations which the author has made, he has no doubt that the second sound is caused by the obstacle which the semilunar valves present to the passage of the blood from the arteries back into the heart, at the termination of the ventricular systole.

At each contraction of the ventricles a quantity of blood is driven by them into the trunks of the arteries, which, being already full, accommodate the addition to their contents by a lateral expansion of their parts nearest to the heart. When the systole of the ventricles is at an end, the elastic force to the arteries, acting upon their contained blood, drives it towards the heart, its entrance into which is prevented by the sudden closing of the semilunar valves: and thus a shock is communicated to the front and upper part of the ventricles, and to the adjacent trunks of the arteries, which may be heard by the ear placed over the region of the heart. The relation, as to time, which the second sound has to the first, its abrupt character, and its coincidence with the end of the ventricular systole, have led the author to adopt the foregoing opinion.

Mr. Carlile then described the experiments from which the greater number of the preceding conclusions have been drawn, and having detailed the circumstances of some made upon living subjects, proceeded to relate those which follow.

1. Artificial respiration having been established in a rabbit which had been strangled, and the heart having been exposed, the following observations were made.

The finger being applied successively to the front, back, and each side of the ventricles, conveyed the sensation of hardness and impulse when the ventricles assumed the globular form, and of softness and flaccidity when they became flattened and expanded. The end of a probe being laid on the front surface of the ventricles, was raised nearly a quarter of an inch during the former of these states, and sank, causing a slight depression on the surface, in the latter. The probe was more elevated when placed on the middle point of the surface, or on the front of the apex, than when placed elsewhere.

The right wing being held aside, so as to admit of the right auricle being seen, this was observed to swell during the continuance of the ventricles in their hardened state, and to diminish its size from the instant in which their flaccidity commenced, its degree of contraction being, however, inconsiderable. The contraction of the appendix was preceded by that of the rest of the auricle, and followed by the instantaneous movement and hardening of the ventricles. The contraction of the different parts of the auricle was successive, commencing as the venæ cavæ, and terminating at the appendix, of which the last contraction was much more sudden and distinguishable than that of any other part.

The heart in this subject continued to beat for an hour, when the motions in all its part ceased, and nearly at the same time; both auricles and both ventricles remaining distended, soft, and full of blood. The heart, separated from the body, was thrown into tepid water, where it remained, soft, and without motion, and had lost the power of contracting itself.

2. A rabbit having been strangled, the heart was exposed while still beating. In about ten minutes the left ventricle ceased to move, and had contracted itself firmly. In a minute or two afterwards all motion was at an end in the left auricle, which was also contracted. The right ventricle continued its movements for forty-five minutes, and during its contraction the apex of the heart was drawn a little to the right side. The right auricle continued to possess motion for an hour and three-quarters; and for the last twenty minutes its contraction proceeded slowly, and with a motion apparently vermicular, over its surface; always commencing at the part contiguous to the venæ cavæ, and ending at the appendix. The right auricle and ventricle contained each some blood when their motions ceased; but the heart having been thrown into tepid water, they gradually expelled their contents, assuming, as those of the left side had done, a firm and contracted state.

The difference of the states in which the hearts were found, after their motions had ceased, in the last two experiments, is remarkable, and appears to admit of the following explanation. In the last experiment, in which no means were employed to continue respiration, the left side of the heart soon ceased to move; because a continuance of the functions of the lungs, as proved by the experiments of Bichat, is necessary to the maintaining of its actions. The firmness of its contraction shows, that although its ordinary motions had ceased, it still retained a considerable share of organic life, as it is known that muscles, whose vitality is quite extinct, have no power of contraction. In the experiment in which respiration was artificially maintained, the left side of the heart continued to beat for an hour, the sustained function of the lungs affording to it a motive for prolonged action; but having been deprived of the influence which the central parts of the nervous system extend to organs in vital connexion with them, its powers of life were exhausted by the long continuance of its motions, and when these ceased, it was quite dead, and incapable of a vital contraction. The right side of the heart in the last experiment seems to have participated in the exhausted state of the left side, because its motions had been performed with much more energy during their continuance than would have been the case had not respiration been artificially maintained.—*Report of the Third Meeting of the British Association for the Advancement of Science.—Amer. Jour. Med. Sci.*

2. *Results of a Series of Experiments in Revaccination, performed in the Royal Army of Wirtemberg.* By Dr. Heim.—The question of the utility of revaccination is now attracting especial attention in Europe, and the materials for settling it are accumulating. In our number for August last, p. 474, et. seq. we presented the result of the revaccination of the Prussian army, and also of the experiments of Dr. Lurott of the Canton Bischwiller, and we shall now offer a condensed summary of the principal statistical and pathological facts collected by Dr. Heim, of Ludwigsburg, Physician to the late Queen of Wirtemberg, who has been officially nominated by the government of that country to superintend the revaccination of the Wirtemberg army. We derive this summary from a paper read to the College of Physicians of London by Dr. G. Gregory, and published in the *London Medical Gazette* of July 12th, 1834.

Prior to the year 1829, it was the customof the Wirtemberg service to vaccinate all recruits who had neither undergone small-pox nor been vaccinated in early life. A variolous epidemic, which showed itself at Stutgard in 1829, occasioned the issue of a ministerial order, dated March 26, 1829, directing that henceforward all recruits should be subjected to vaccination who could not show satisfactory *cicatrices* either of small-pox or of the vaccine. In the autumn of '32, small-pox broke out in the garrison town of Ulm; and, again, a third epidemic occurred at Ludwigsburg in 1833. These repeated visitations of small-pox in the Wirtemberg territories occasioned the issue of another order, dated February 7th, 1833, directing indiscriminate revaccination of all recruits, without reference to vaccine cicatrices.—Directions were further given for revaccinating every individual of the two garrisons of Ulm and Ludwigsburg, of whatever age or standing in the army—those being the towns in which the variolous epidemic had displayed itself in the greatest force.

The general results of these very extended trials are given by Dr. Heim in two tables.

Table No. I. gives a general survey of the success attending the revaccination of the entire garrison of Ludwigsburg during the summer of 1833. The garrison consisted of a regiment of artillery, a regiment of infantry, and two regiments of cavalry: total, 1683. Of these there were revaccinated, with the best success, 577, (one-third of the whole)—with modified or partial success, 366—without success, 740. Nearly the whole of the subjects of these experiments were, as might be expected, adults, between twenty and thirty years of age, and of course the greater portion of them had been vaccinated in infancy. 144 only were above or under the ages now specified.

Of the 577 men revaccinated as above stated, with the best success, 293, (more than one half,) had perfect cicatrices. On the other hand, out of the 740 revaccinated *without* effect, 222 had imperfect scars, and 136 retained no mark whatever of their first but effective vaccination.

Table No. II. presents a general summary of the results of the revaccination of the *Recruits* of the Royal Army of Wirtemberg, from 1829 to 1833, of which the following appear to be the most important:—

| | |
|---|---|
| Total numbers upon whom the operation of revaccination was performed - - - - | 4802 |
| Of them were revaccinated— | |
| With normal or perfect success - - - | 1208 |
| With modified or imperfect success - - | 956 |
| With success, but in a degree not accurately specified | 914 |
| Without success - - - - - | 1724 |
| Total - - - | 4802 |

If we deduct from the total number revaccinated, 4802, those in whom the success, though perhaps in the pleurality normal, is yet not pointed out with precision, (914,) the proportion of those revaccinated with good success is 30 per cent.—of those with modified or partial success, 24 per cent.—of those without result, 46 per cent.

The same table presents us with a series of data from which the value of the cicatrix, as a test of constitutional safety, may be inferred.

Of 4111 persons revaccinated, 2193 had normal cicatrices, In 2390 cases the operation produced a certain degree of effect. One-half passed through the second vaccination in a normal manner; the other half imperfectly, and in a modified form. Of the former, or regular revaccinations, (1208 in number,) 664 had perfect cicatrices; that is to say, one-half of these upon whom the process of revaccination produced the most *decided* effects, had scars, from which an *opposite* result might have been anticipated.

The converse of the proposition leads to a like conclusion. As thus:—Of 1722 persons on whom the operation of revaccination produced *no* effect, and in *all* of whom, therefore, good cicatrices might have been expected, 500 had *imperfect* scars, and 259 bore no scars whatever.

Again: of 956 persons in whom, upon being revaccinated, the cowpock appeared, although in a modified form, 572 bore upon their arms *good* cicatrices, 278 had *imperfect* cicatrices, and 104 had *no* cicatrices whatever.

From these and various other facts Dr. Heim concludes, that the inspection of the scars left by the primary vaccination is devoid of all practical interest: in other words, that no satisfactory conclusion, as to the resusceptibility of the disease or otherwise, can be derived from such a source.

Such are the principal results deducible from the tabular statements transmitted by Dr. Heim. The learned author appends to these certain pathological observations made by himself while superintending, in his official capacity, the revaccination of the garrison of Ludwigsburg, in 1833.

The first, and certainly the most interesting circumstance adverted to, is the effect which the extended system of revaccination pursued by the authorities of Wirtemberg had in checking the variolous epidemic. His words are:—"This powerful revaccination of the men comprising the garrisons, (of Stutgard, Ulm, and Ludwigsburg,) without reference to the quality of the cicatrices, destroyed the spreading of small-pox among the soldiers." In another part of the MS. he uses these expressions:—"In this manner the revaccination passed in a few weeks through all the regiments of the garrison, and the spreading of the epidemic small-pox among the military was set real bounds to by it."

The next circumstance adverted to by Dr. Heim is entirely new to the practitioners of this country, namely, the difference of result according as the propagating lymph was derived from the arm of a child or that of an adult. It has long been known that children are vaccinated most successfully from others of their own age. Dr. Heim's experiments lead him to believe, that *adults* are vaccinated most effectually from the vesicles *of adults.*

His words are these:—

"In the first regiment of cavalry many fruitless, or at least unsatisfactory, trials were made with fresh and good matter taken from the arms of children. At length I made it prosper upon a soldier, in whom fifteen exquisitely perfect pustules were produced. By revaccinating with this *adult lymph*, I succeeded with those in whom vaccination from the arm of the child had *failed*. Even in individuals of a higher class of society, with all favorable qualities for good success, the trial of vaccination from the child's arm frequently failed, while I soon succeeded in it from the arms of adults."

That this was owing to some peculiarity in the law of *adult vaccination*, and was not attributable to any fault in the vaccine matter of the infant, was proved by its *constant* success when infants alone were the subjects of trial.

Further on, Dr. Heim thus expresses himself:—

"Of the superior effect of vaccinating from *adults* to *adults*, I have had experience for the last six years, but the opportunity which has been recently offered of trying it on a large scale, has been rewarded *with the most splendid success*....My colleagues, (he adds,) of this place, (Ludwigsburg,) have also made the same observations.

In another part of the memoir, while adverting to the employment of adult lymph, to which he attributes the superior efficacy of the revaccinations of 1833, Dr. Heim notices the prejudices that so generally exist in Germany as well as in England, against employing the vaccine matter of adults in propagating the disorder. "This false opinion," he states, "is the more striking as it is neither defended by obvious reasons, nor confirmed by experience. On the contrary, there are cases known to us where the modified vaccine of revaccinated persons produced the *genuine* vaccine in children not previously vaccinated: nay more, in some instances it took effect upon those I had vaccinated more than once, but without success, using recent lymph from the arms of children."

Having satisfied himself of the truth of his position, that important practical differences do exist between adult and infantile vaccination, Dr. Heim proceeds to reason upon it.

"We might expect," he says, "à *priori*, that the vaccine lymph of adults should be more *energetic* than that of children, for the same reason that we find measles, scarlet fever, and even small-pox itself, to be more *dangerous* in adults than in children." He next throws out the very ingenious hint, that it was to the employment of vaccine matter from the arms of *adults*, that Jenner was indebted for the splendid success which attended all his first trials with the vaccine. Dr. Heim evidently considers this as the chief feature of his memoir, and he invites the attention of physicians to it in an especial manner, as to a subject of great scientific and practical interest.

Dr. Heim supports the doctrine of a gradual return of the vaccine susceptibility in persons even the most effectually vaccinated in early life, but his further speculations on this subject are confused and scarcely intelligible.

Dr. Heim has made no experiments with the variolous contagion, so as to determine positively, whether the susceptibility of small-pox and the resusceptibility of cow-pock acknowledge the same law.— This important branch of the subject requiring, on the part of the experimenter, such extreme caution, must be left to be determined by future inquirers. Dr. Heim indulges the notion, that the resusceptibility of small-pox and the vaccine do *not* coincide, but he throws out the suggestion without offering any adequate proofs in its support.

Dr. Heim details a curious case in which small-pox and measles occurred simultaneously, and he seems to hint that there is some sort of analogy in the relations of cow-pock to small-pox, and of small-pox to measles.—*Amer. Jour. Med. Sci.*

3. *On the Use of Colchicum Autumnale in Leucorrhœa.*—GEORGE RITTON, Esq. in a communication in the *London Lancet* of 2d of August last, states that he has treated a vast number of cases of leucorrhœa with the powdered root of colchicum with almost invariable success. He commences its use with grains of the powdered bulb made into a pill with hard soap, to be given three times daily, and the dose increased to five grains. During the period the patient is taking the colchicum, it will be absolutely necessary, Mr. R. says, for her to abstain from *every* beverage which contains alcohol. Five grains of powdered bulb of colchicum, exhibited three times daily, will very generally cure leucorrhœa in ten days. Some cases require its continuance for three weeks, and others for a month. The discharge sometimes returns after the discontinuance of the medicine, but after further perseverence in its use Mr. R. has finally found it almost always successful.—*Ib.*

4. *Treatment of Sore Nipples by Nitrate of Silver.*—Chaps, abrasions, and inflammation of the nipples in nursing women, are productive of extreme suffering. The following method of treatment is asserted by Dr. A. J. HANNAY, in a communication in the *London Medicnl Gazette* of 9th of August last, to never fail to afford relief, and ultimately effect a cure. "Having gently, but carefully, dried the nipple, touch it freely with a sharp pencil of nitrate of silver. Be sure to insinuate the pencil into the chaps or chinks; then wash the nipple with a little warm milk and water. In most instances, the pain, though smart at first, soon subsides, and a little simple ointment, or one made with the flowers of zinc, is all that is requisite to heal the sore. I occasionally wash the nipple with the saturated solution of borax, before and after the suckling the infant. Some suffer a great deal of pain from the application of the caustic, this must not be heeded. A draught, containing an opiate, such as sol. mur. morph. thirty drops, soon brings relief, and the part is presently easier. Some require to be touched more than once,—nay, several times; but each succeeding time it is less painful. I have heard of a solution of nitrate of silver being tried: I can positively assert that it is inferior to the solid caustic, both in relieving and healing these painful affections."—*Ib.*

5. *On some of the Effects of the Secale Cornutum.*—The third No. of the fortieth volume of *Rust's Magazin*, contains an interesting memoir by Dr. MULLER on the above subject. The author does not entertain any fears of the accidents, said by some practitioners to result from the secale cornutum given to hasten delivery, as he has never seen a case in which it has been hurtful to either mother or child, when the delivery was at full time. Dr. M. has repeatedly used the article to produce abortion in cases of hæmorrhage occurring at the second, third, fourth, or even at a more advanced stage of utero-gestation, and in every case the desired effect was promptly produced, and without hæmorrhage. In most of the cases, other abortives had proved inefficacious, and the least delay would have been fatal. Dr. M. has also resorted to the ergot to promote the expulsion of the placenta, and also to arrest uterine hæmorrhages after both natural and artificial delivery, after abortion, those which result from external violence, and in menorrhagia; also in all kinds of pulmonary hæmorrhages; and finally, in nasal and intestinal hæmorrhages, and he thinks that no other means is more useful. It is, especially, however, in uterine hæmorrhages following delivery or abortion, in menorrhagia and pulmonary hæmorrhages that it is most efficacious. Professor Bazzoni has published eight cases of leucorrhœa cured by this remedy; and Dr. M. says that he has long used it in the same disease with constant success, and also in blenorrhagia in men, and particularly gleet, which has resisted every other treatment. Dr. M. considers the best mode of administration to be in powder freshly prepared, and mixed with sugar. He gives it in doses of from five to ten grains every two hours. —*Amer. Jour. Med. Sci.*

6. *Sulphuric Acid as a Prophylactic against Saturnine Colic.*—Some time ago M. Gendrin proposed sulphuric acid lemonade as a prophylactic against, as well as remedy for saturnine colic, (see this journal for May, 1832, p. 241.) M. G. has recently communicated to the Academy of Sciences, some additional interesting information on this subject. He states, that M. Rouard, director of a manufactory of white lead, caused all his workmen to take the sulphuric acid lemonade, and from that moment lead colic ceased to occur among the men in the establishment. During two months, only four workmen were slightly affected with lead colic, and this exception is explained by circumstances peculiar to these workmen. But, he adds, that at the same time that these results have confirmed the utility of the measure as regards colic, six of the workmen were attacked with symptoms hitherto regarded as the effects of lead colic, as cramps, muscular debility, and nervous epileptic symptoms. These symptoms, M. G. ascribes to a layer of oxide and carbonate of lead, combined with the epidermis. This observation he considers accounting for many symptoms hitherto difficult of explanation, and points out the origin of the relapses and symptoms which so often supervene after the cure of colics in workmen habitually exposed to the powerful action of the causes of the disease. He further adds, that whether the sulphuric acid be resorted to as a prophylactic or remedy, it must be employed externally, as well as internally. Conformable to this indication, the workmen in the establishment of M. Rouard, at the same time that they take the sulphuric acid lemonade as drink, use lotions of the same to the surface of their bodies.—*Ib.*

7. *On Creosote.* By M. Reichenbach of Blansko.—M. Reichenbach, to whose labours we owe the discovery of Paraffine, of Eupione, and Picamore, has recently found, in the products of the destructive distillation of wood, a new substance which he calls Creosote, from the Greek words *kreas*, flesh, genitive by contraction *kreos*, and *sozo*, I save.

This substance is highly interesting, not only on account of its chemical properties, but from its useful application to therapeutics, domestic economy, and the preservation of provisions for long voyages. Two processes are given for its preparation. By the one, the creosote is obtained from pyroligneous acid; by the other, from the tarry matter which distils over along with that acid. These processes do not differ much; both are tedious, but the latter method seems to be the easier. The tarry matter yields an oil by distillation, to which, after being rectified and heated, carbonate of potash is added, to neutralize the acetic acid associated with it. The acetate of potash separates, and the oil is again distilled, care being taken to reject the first products, and not to carry the distillation to dryness. The oil that comes over is then treated with a solution of caustic potash of sp. gr, 1.12, great heat is produced, and a portion of eupione, &c. formed, which floats on the surface. These are rejected, and the alkaline solution is slowly made to boil in an open vessel. A chemical action takes place,—it absorbs oxygen from the air, and assumes a brown colour. After it is cooled in the open air, diluted sulphuric acid is added until the oil is set free. It is again distilled with water, to which a little caustic potash should be added. The oil is then separated from the water in the receiver, and again treated with a solution of potash, sp. gr. 1.12, boiled as before—cooled—treated with rather an excess of sulphuric acid—poured off from the sulphate of potash—well washed with water to carry off the excess of acid,—again distilled with water, to which a little phosphoric acid is added, to saturate the ammonia associated with the oil. Lastly, it is dissolved in caustic potash, and if the preceeding operations have been carefully attended to, the creosote and the potash unite, and the mixture, when heated, leaves no residuum of eupione, nor becomes brown by exposure to the air. The creosote may then be separated from the potash by distillation, and, although not quite pure, is sufficiently so for medical purposes. The foregoing is a very imperfect outline of the process, which will be seen to be sufficienly tedious. The processes will be found minutely described in the Annals of Schweigger-Seidal, Vols. VI. and VII.

Creosote is an oily, colourless, transparent liquid, possessing great refrangibility. Its odour is penetrating, disagreeable, and similar to that of smoked beef. It is of the consistence of oil of almonds, and has a sp. gr. of about 1.037, at 20 deg. Cels. (68 deg. Fahr.) It boils at 203 deg. Cels. (397.4 deg. Fahr.) and is not congealed at a temperature of —27 deg. Cels. (—16.6 deg. Fahr.) It burns with a smoky flame. It is a non-conductor of electricity. At 20 deg. Cels. (68 deg. Fahr.) it forms with water two different combinations, the one containing one-fourth part of creosote in 100 parts of water, the other, ten parts of water in 100 creosote.

This substance forms numerous interesting compounds, with acids and alkalies. Concentrated, it dissolves the deutoxide of copper, and assumes a chocolate-brown colour. At a boiling heat it reduces the deutoxide of mercury, and is then transformed into a resin, which has no longer the properties of creosote. Nitric acid acts on it strongly, and acid vapours are disengaged. It combines with chlorine, bromine, iodine, phosphorus, and sulphur. Potassium thrown into it disappears, gas is disengaged, and potass remains combined with thickened creosote. From this combination the creosote separates by distillation. Concentrated sulphuric acid added in small quantities gives to creosote a reddish colour; but when the quantity of acid is increased, the creosote becomes black. Of all the organic acids, the acetic seems to have the greatest affinity for creosote, uniting with it in every proportion.

This substance, when cold, forms two combinations with potash.—The one is an anhydrous liquid, of an oily consistence; the other is a hydrate, and crystallizes in white scales. All the acids, not excepting carbonic acid, separate the creosote from these combinations.—With soda, it forms combinations similar to those with potash. It has a great affinity for lime, and the hydrate of barytes; with these bodies it forms compounds of a dirty white colour, soluble in water, but which, when dried, assume the appearance of a rose-coloured powder.

Creosote, in a warm and cold state dissolves a great number of salts. Some are reduced, but the greater part are separated in the form of crystals by cooling, such as the acetates of potash, soda, ammonia, lead and zinc, and the hydrochlorates of lime and tin. It reduces the acetate and nitrate of silver.

Alcohol, ether, acetic ether, carburet of sulphur, eupione, and oil of petroleum, combine with creosote in every proportion. Paraffine, though issuing from the same source with creosote, has little tendency to combine with it. Indeed, the combination cannot be effected, unless eupione be present, and in a direct ratio to the quantity of eupione. Creosote with difficulty dissolves caoutchouc, and only by the assistance of boiling, differing very much in this respect from eupione, which readily dissolves caoutchouc.

If to a solution of albumen, in a large quantity of water, a single drop of creosote be added, the albumen is immediately coagulated. When fresh meat is put into a solution of creosote, allowed to remain for half an hour, or an hour, then withdrawn, and afterwards dried, it may be exposed to the heat of the sun without putrefying, and in the space of eight days it becomes hard, the colour changes to a reddish-brown, and the flavour is that of good smoked beef. Fish may likewise be preserved by it. It is pretty evident that creosote is the anti-putrescent principle of pyroligneous acid and of wood smoke.

M. Richenbach has ascertained that creosote does not act on pure fibrin, which by itself is said not to be susceptible of putrefaction.—Its action upon the animal economy is deleterious. Placed upon the tongue it occasions violent pain, and when poured, in a concentrated state, upon the skin, it destroys the epidermis. Insects and fish thrown into it immediately die. Plants also perish when watered with it. M. Reichenbach has made experiments with this substance concentrated and diluted, and his success has surpassed his expectations. It has, he alleges, effected a speedy cure in cases of caries, of cancer, and of carcinomatous ulcers.

M. Schweigger-Seidal has made a comparative examination of creosote, and the aqua Binelli, from which he has come to the conclusion, that the fundamental base of this hemostatic liquor is creosote, of which it is only an excessively weak solution.—*Edin. Med. and Surg. Jour. Jan.* 1834.—*Ib.*

8. *New Method of Preparing Creosote.* By M. Calderini, apothecary at Milan.—The essential oil obtained by the destructive distillation of wood, is to be put into an iron vessel, and exposed to a gentle heat. The vessel is then to be taken from the fire, and slaked and sifted lime to be poured into it little by little, and with continual agitation, until the effervessence ceases, and the mixture becomes a hard mass, which is to be allowed to cool, and then powdered. A cast iron retort is to be two-thirds filled with this powder, and placed in a reverberatory furnace. A receiver is to be fitted to the retort at the moment when the white vapours which first come over become yellowish. The distilled liquor is to be placed in a filter of paper moistened with water, to permit only the aqueous part to pass, and the oil left is to be washed with pure water, which is to be allowed to filter. The oil thus washed is to be placed in an iron vessel, and aqua petassæ of sp. gr. 1.125 is to be added in the proportion of three parts to two of the oil. The mixture is then to be boiled for a moment with a gentle heat, after which it is to be taken from the fire, allowed to cool, filtered and mixed with dilute sulphuric acid, till it becomes slightly acid.—The mixture is then to be left at rest, and an oily matter will be found floating on the top, which is impure creosote. This is to be collected, washed on a filter, put into a glass retort, placed in a sand-bath, and distilled. The first portion is to be laid aside, and what comes over afterwards of a pale yellow colour when heat is added, is creosote.—The distillation is to be stopped when the drops become of a deeper colour. If the distilled creosote be not sufficiently pure, it is to be dissolved in the *aqua potassæ*, and treated as before, always rejecting the first and last parts that come over on distillation, and this process is to be repeated until it becomes perfectly pure. When the creosote is obtained pure it is to be kept in well-stopped bottles. It is known to be pure when it is colourless, transparent, of specific gravity 1.037, and possessed of great refrangibility. If a drop be placed in contact with the white of an egg, it is suddenly coagulated. If it be dissolved in a small quantity of *aqua potassæ*, the solution, when heated in contact with the air, does not assume a brown colour, as happens when the creosote is impure, but becomes slightly reddish.—*Ibid. Oct.* 1834.—*Amer. Jour. Med. Sci.*

9. *Medicinal Properties of the Creosote.*—From all accounts the creosote seems to be a potent, and consequently, under a judicious use, a useful remedy. A drop put upon the tongue causes very severe pain, and blisters the part; even the epidermis is usually detached from a portion of the skin, moistened with the pure creosote.

The solution of it in water is the preparation which Reichenbach has chiefly employed;—its strength may be made to vary according to the strength of the irritation required; but in most cases a solution of one part of the creosote in fifty of warm water will be found most convenient. He has used it successfully in scalds and in burns, whether the epidermis has been detached or not; in numerous cases of chronic herpes and impetigo, in itch, &c. When these cutaneous diseases are very obstinate, and resist the effects of the creosote solution, he is in the habit of applying the substance either directly and in its pure state, or mixed with lard, so as to form an ointment;—under the use of this ointment, the pustules or vesicles very quickly dry and fall off. The period usually required for the cure varies from one to three weeks; and as a matter of course, this must depend on the duration of the disease, and on the constitution of the patient. Several cases of troublesome, and sometimes apparently malignant ulceration are reported as having been cured by the creosote. Scrofulous ulcers are benefited by it in an especial degree; and when there are any sinuses or fistulæ, no injection will be found so useful as water impregnated with the creosote. If the ulcers should be obstinate, it may be well to touch the edges of them with the pure creosote; but in most cases the application of pledgets of linen wet with the solution will be found sufficient. Tooth-ache may be often cured instantaneously by introducing a drop of it into the cavity of the decayed tooth. Even the mere gargling with the solution in water, will not unfrequently relieve the pain. The efficacy of the use of the creosote internally we should deem much more problematical, and especially in such a disease as hæmoptysis, for which it has been recommended; the dose given was four drops rubbed up with lump sugar, (it is not stated whether the four drops are to be given in one or in divided doses,) and this was repeated for six or seven days.—*Bulletin Therapeut.—Med. Chir. Rev.—Bost. Med. Sur. Journ.*

10. *Preservation of Leeches.*—M. Bertrand recommends that leeches after they become detached from a part should be gently passed between the thumb and fingers (or as it is vulgarly called, stripped) so as to make them disgorge most of the blood which they have sucked, and then be put into water moderately sweetened.

In this method leeches, we are told by M. B. may not only be kept for many years, but will retain their activity, and be fit for reapplying every third or fourth day.

Dr. Scheel has more recently recommended the following treatment as still more effectual.

The leeches should first be put into a warmish plate, and be well besprinkled with carbonate of soda; when they have disgorged most of their blood, they should be washed several times with tepid water, then put again upon a plate and some sugar sprinkled upon them; and lastly, be washed with cold water and put into a large vessel full of it, gently sweetened.

The sugared water must be changed once a day, or oftener, if it becomes discoloured. Those leeches which die or even become wrinkled and faded, should always be cast away.

Dr. S. has succeeded in preserving leeches thus for a great length of time, in good health, and in a condition ready for use every six or seven days.

When there is a very great dearth of leeches, the following expedient may be had recourse to. With very sharp scissors we may cut them right across, somewhat nearer the tail than the head, and then apply them as usual. As the animal sucks, the blood trickles from the divided end. As a matter of course, the attendants must be on their guard to prevent an excessive discharge by detaching them at a proper time. They are then to be gently squeezed, and put into fresh water. If the divided extremity should become agglutinated together and closed, we must employ a rather firmer pressure from the mouth downwards, so as to cause the blood to force an exit. Leeches which have been mutilated in the manner above mentioned, have been used several times every day for many weeks.—*Med. Chir. Rev. and Algemeine Medic. Zeitung.—Ib.*

11. *Action of rarified or condensed Air on the surface of the Body.*—M. Junod has read a memoir to the Institute of France on the effects of the condensation and rarefaction of the air on the whole body or on individual members. The apparatus he uses is a spherical brass receiver, three metres, three decimetres in diameter, the sides of which are maintained within by two iron rings supporting a bench on which the subject of the experiment is seated. Light is let into the apparatus by two glass plates in the sides. The upper part has an opening which may be hermetically closed by means of a spherical plug, or spherical leather cup. This cover has three appendages, intended to contain a thermometer, a barometer, or manometer, and the third a cock for the renewal of the air. The condensation and rarefaction of the air is effected by a pump communicating with the interior of the sphere by a tube.

If the atmospheric pressure is augmented by one half, the phenomena observed are:—

1st. Sensation of pressure on the tympanum which is driven inwards: this gradually disappears as the equilibrium is re-established.

2nd. Easier play of the respiration, deep and less frequent inspiration: in a quarter of an hour agreeable warmth of the chest, as if the pulmonary areolæ, hitherto incapable of respiration, dilated to receive the air.

3rd. Pulse somewhat frequent, full, and not easily compressed: the superficial veins flaccid and even completely obliterated, so that the blood seems to return to the heart by the deep seated veins.

4th. Functions of the brain more active, the imagination vivid, &c.; in some a sensation of drunkenness.

5th. This increase of inervation affects the muscular system, the movements of which are easier and more precise.

6th. Digestive functions more active: no thirst.

7th. Salivary, venal and grandular secretion in general more copious.

When the atmospheric pressure is diminished by one quarter, the inverse of the above are observed; that is, distention of the tympanum, producing a sensation resembling pressure, which is also dissipated by the renewal of the equilibrium; impeded respiration, short and frequent inspirations, often dyspnœa, pulse full, compressible, frequent; turgescence of the superficial vessels of the eyelids and the lips; frequently hæmorrhage with tendency to syncope, disagreeable heat of the skin, the functions of which are increased in activity; enfeebled inervation and muscular power, diminution of the grandular secretion.

Pressure on one member increased by one half is effected by a peculiar instrument and produces these effects. The skin becomes pale, the superficial veins become flaccid, the limb is diminished in volume, the circulation is suspended over a varied space. After the operation the limb is sensibly lighter, the movements easier and more precise. Other phenomena occur according to the limb operated upon. Thus, if one of the legs be acted on, vertigo, tinnitus of the ears, sparks of the eyes, difficult respiration, &c., supervene.

Pressure diminished by one-ninth on one or more limbs gives these symptoms. The skin is distended and colored, the limb increased in volume in a very few minutes, the temperature of the surface reaches that of the deep-seated parts; copious transpiration forming a vapor which condenses on the sides of the apparatus. After the operation a slight numbness of the limb is felt.

When pressure is diminished on one or several limbs the following general phenomena are seen. The head is light, the face pale, the pulsation of the facial branch of the frontal artery becomes slower, thready, and is sometimes lost: there is then tendency to syncope. The respiration is easier, the digestive functions lose their activity; nausea sometimes comes on; towards the end of the operation the traspiration, which had been confined to those parts within the instrument, becomes general.—*Lond. Med. and Surg. Journ. Nov.* 1834.—*Archives of Med. and Surg. Sci.*

12. *Re-union of the external Ear after it had been completely severed.* By D. Mariani.—This was the case of a man, who, in a house of bad repute, had his ear severed in an affray. He carried it away with him, rather to conceal his accident than on account of pain. Dr. Mariani, to whom he applied, resolved to endeavor to obtain the re-union of the separated portion. With this object, having first washed it in diluted alcohol, and clipped away some shreds, he re-applied it, and by means of four sutures, and a piece of gum elastic fixed in the meatus of the ear, secured it in its natural situation. The adjustment was still further secured, by means of adhesive strips and a dandage. He had no hope of obtaining union between the cartilaginous structures, but thought that the soft parts might unite.

On the next day he removed the dressings, to see that no displacement had taken place, when he discovered with pleasure, the existence of a red suffusion along the edge of the wound. The individual was affected with fever, thirst, and pain of the head. On the eighth day these symptoms had subsided, and the fragment of the ear had resumed its natural temperature. The extremities of the incision were the first to unite, and that connected with the lobe first of all. The other parts assumed a sloughy condition, and did not unite until the cartilage became covered with granulations. The cure was completed in about a month, and all that remained was a linear cicatrix which was almost imperceptible.—*Filiatre Sebezio di Napoli. Maggio.—Archives Med. and Surg. Sci.*

13. *Identity of Gonorrhœa and Syphilis.* By Professor HUFELAND.—"Gonorrhœa arising from infection is always syphilitic, but is modified, and rendered less infectious by the secretion poured out by the mucous membrane of the urethra." This proposition which was advanced by the author thirty years ago, he thinks has been confirmed by long experience.

The following are the arguments by which it is corroborated:—

1. *The same cause.*—Two individuals may be infected by one person,—the one may have gonorrhœa,—the other a syphilitic chancre.

2. *The same effects.*—A person affected with gonorrhœa may communicate syphilis to another, or infect himself with that disease. Gonorrhœa matter introduced into the eye may give rise to a venereal ophthalmia; or a gonorrhœa too suddenly suppressed may be followed by bubo, chancre, condylomata, &c. and daily experience proves, that fluor albus of the female, which supplies the place of gonorrhœa, is capable of occasioning syphilis.

3. *The same treatment.*—Although the professor acknowledges that a majority of cases of gonorrhœa are cured either by the powers of nature, or antiphlogistic treatment; he affirms, that when this is not the case, and pains takes place in the urethra, with inflammation in the throat, and other consecutive symptoms, calomel is always the most efficient remedy.

The difference between the virus of the two affections is, that, of gonorrhœa is incorporated as it were with the mucous secretion, and acquires thereby a milder character, so that it may lose its infectious quality both as regards a second person and the individual himself. It is besides sometimes thrown off with the mucous secretions. The virus of a chancre, on the contrary, is more active and corrosive, for the same reason that corrosive sublimate, or any other poison, introduced into the system in its isolated state, is more energetic than when enveloped in mucus.—*Journ. for Praktisch Heilkunde,* 1834.—*Gaz. Medicale.—Archives Med. and Surg. Sci.*

14. *Treatment of Pulmonary Consumption by Inhalation.* By SIR CHARLES SCUDAMORE.—I regret that a great deal of scepticism still appears to prevail as to the merits of inhalation, and especially as regards phthisis pulmonalis, for, I believe, its utility is not questioned in chronic bronchitis, and in certain forms of asthma.

Some are disposed to throw ridicule, and a few even calumny, on any professed attempt to cure tubercular consumption; but such adversaries do not deserve one moment's consideration. We owe it equally to science and to humanity, to make unceasing exertion to lessen, to the utmost of our power, the influence of this destructive and fatal scourge.

A few call in question the propriety of employing any active agent to be administered by inhalation, alleging that the danger of producing injurious irritation to the air-passages and the lungs is more certain than the chance of removing the tubercular disease. The fact, however, is, that in no form of pulmonary complaint does inhalation relieve with so much promptitude and certainty, as in chronic irritation of the bronchial mucous membrane; and with respect to the tubercular irritation, I am willing to rest the whole proof of my recommendation upon actual experience, and the large share of success which I have obtained.

It unfortunately happens that a great proportion of cases of phthisis pulmonalis have acquired so much inveteracy as to be incurable, when they are first presented to the physician's care. Too probably, little or no attention has been paid to the first inroads of the disease; it makes progress with sure but insidious march; and when, at last, an anxious appeal is made for medical exertion, the lungs may have undergone such extensive disorganization as to allow of no other possible relief than some mitigation of the symptoms.

Failure of success on such occasions ought not to impair the credit of the mode of treatment which I am advocating; but I am too well aware that this serious disadvantage to the *good character* of inhalation is always more or less incurred when the case ends fatally, notwithstanding that only slight, if any, hopes of success might have been held out to the friends of the patient; for with regard to the patient himself, we must sustain his mind by kind encouragement. The despondency of the invalid would greatly diminish our power of affording relief.

The most frequent cause of regret which I have to experience, is the delay of the patient, or of the friends of the patient, to make a timely application for the material benefits to be afforded by inhalation. There is, undoubtedly, a satisfaction in mitigating sufferings, but yet the task is a melancholy one of watching the progress of a disease which has become incurable.

For the better comprehension of my statements in the following cases, I take the liberty of referring the reader to the second edition of my work, in which I have fully detailed my principles and method of treatment.

Case I.—J. A., æt. 47, a superintendent of a gas district, tall and well-proportioned; of delicate constitution. His father and mother, and uncle, died from consumption. He consulted me in January, 1834. He was very thin, and stated that "he had lost almost all of his flesh and strength, and feared he was past help." He had been ill with cough and shortness of breath since the beginning of December, having caught cold from continued exposure to a N. E. wind. I found his pulse from 96 to 106, and feeble; the respiration was distressingly hurried by slight exertion; the animal heat was 101 degrees. By auscultation, great obstruction to the breathing was shown, and the sound on percussion was dull, these indications being most remarkable on the right side. The sputum was greenish in appearance, not copious, and, examined by the optical experiment, did not present the prismatic colours; the tongue was morbidly red; the appetite impaired; the bowels prone to diarrhœa. He had sometimes two hectic paroxysms in the day, and always one; night perspiration was more or less abundant, and he never obtained comfortable sleep.

I directed the inhalation of a solution of iodine, with the addition of a saturated tincture of conium (for the formula see my second edition,) three times a day; the internal use of sarsaparilla and alkali twice in the day, and at night the acetate of morphia, with diluted sulphuric acid and syrup of tolu. The chest was washed night and morning with the lotion of purified pyroligneous acid, eau de Cologne, and water, used just tepid, followed by the use of the flesh-brush. The diet was nourishing and supporting.

The means of treatment agreed; but as I was convinced that the lungs were obstructed by tubercles which would soften, I viewed this as a case which, in the most favorable event that could happen, would become much aggravated before any convalescence could take place. In other words, he would get worse before he could get better.

From taking cold he lost his smell and taste, and suffered from a subacute attack of pleurisy, which was removed by blistering. He described that he felt very sensible relief from inhaling, and, together with the good effects on his chest, he was sensible of improvement of appetite from it. The intestinal canal had for some time been very irritable and often painful. He received benefit from a mixture or infusion of catechu with mucilage, tincture of kino, compound chalk powder, and Ford's laudanum, (a saturated tincture of opium with the addition of spices.) He took occasionally at bed-time, when there was evidence of vitiated biliary secretion, small doses of the *hydr. c. cret.*, joined with *c. pulv. ipecac. compos.*

A journal of this case would occupy too much space; I shall content myself therefore with a brief general statement. At the middle of March it was evident, both by the general signs and by the local indications, that the expected softening process in the tubercles was advanced. He was thinner and weaker, and his ankles were swollen towards night; the expectoration was increased in quantity, more or less puriform, and often colored with blood; the night sweats were usually profuse. In the right axilla I detected by the stethoscope a resonance approaching to pectoriloquism. The sound on the right side was dull to a great extent, and the breathing was very imperfect.

For the most part he maintained a tolerable appetite, but his appearance was altogether so unpromising, that I almost despaired of any success. He persevered with inhaling. I changed the iodine for chlorine for a short time, but of his own accord he returned to the use of the iodine, judging from his feelings that it was by far the most useful. When the bowels became settled, I gave him the *mistura ferri composita*, and changed it at the end of a fortnight for the mixture of sarsaparilla with alkali, or for one with quinine, according to circumstances. I directed some porter and a little sherry, with as substantial and nutritious diet as his digestive powers would allow.

At the end of another fortnight pectoriloquism in the right axilla was unequivocal; the expectoration was abundant, and the sputum examined by optical means gave prismatic colors. The respiration was improved; there was less of hectic fever in the day, and the night perspirations more abated.

From this period he improved, steadily pursuing all the means of treatment. Now, in the third week of November, he does not appear the same individual as when he first consulted me, so wonderfully is he recovered in flesh and strength, and his spirits are quite regained. He has scarcely any cough remaining, and his breathing is comfortable. The pulse is 76 to 80; the animal heat 98 degrees. He has resumed his former occupation without inconvenience, and I have every expectation that with proper care on his own part, he will enjoy future health in a very comfortable degree.

Case II.—A gentleman, aged 26, of the middle height, muscular, of the mixed temperament, well formed in the chest, usually enjoying good health, with the exception of a liability to take cold, and have a catarrhal cough in the winter season, caught cold from exposure for some hours on horseback to a north-east wind, in March, 1833. Inflammatory symptoms, with pleuretic pain occurred, and general and local bleeding was used, with blistering, and an antiphlogistic treatment.

I saw him first in the beginning of June, and received the following account of his case from Dr. Skrimshire, of Peterborough, in Northamptonshire. "This patient is the subject of recent but rapid tubercular phthisis." Then detailing the treatment which had been used, he adds, "I have not, however, at any time reduced the rapidity of pulse, or the urgency of the cough, for more than a day or two; the wasting has been progressive and rapid; and the expectoration, though never profuse, has for the last three weeks or a month been puriform."

I found the dull sound on percussion over a considerable extent of the left side; the respiration imperfect, and near the axilla the indication of pectoriloquism was sufficient to render it probable that a small cavity existed at the upper part of the lung. The signs on the right side were good. His breathing was much hurried on slight exertion. The cough was harrassing: the morning expectoration was considerable, creamy, of disagreeable odor, and gave prismatic colors. He was suffering from slight pleurisy of the left side. He could not sleep without having the head much raised, nor lie well on either side. He had been more sensible of daily hectic fever and night perspirations a month before, then at the period of my visit. The pulse ranged from 112 to 120; the animal heat was 101 deg. He had greatly lost flesh and strength, and his pale and hollow cheeks proclaimed at once the severe character of his disease. It was encouraging, that his appetite was for the most part good, and that the digestive functions were not much disturbed, but the urine deposited lateritious sediment abundantly. So soon as I had removed the pleuritic pain by local treatment, I directed the inhalation of iodine with conium, and treated him altogether on the principles which I have detailed in the statement of my other cases.

This gentleman improved so regularly and favorably, that he went into the country at the end of July, with the feelings of nearly restored health. He had gained flesh and strength, and was almost free from cough. The pulse was regularly under 80, and the animal heat was reduced to 97. He extolled the inhalation as the great source of his cure.

Since this period my patient has taken a journey of pleasure to Paris, and I have the satisfaction of hearing that he finds his strength and general health re-established.*

Case III.—A young gentleman, aged 22, tall, slight, with circular chest, of the nervous temperament, while laboring under great mental excitement, which was quickly followed by inflammation of the membranes of the brain, exposed himself, in a state of delirium, to the cold night air, when without clothes. Bronchitis followed. He lost blood from the arm and by leeches, and blisters were applied.—It was observed that the severe symptoms affecting both the head and chest alternated remarkably. When I first visited the patient, he appeared pallid and exhausted, scarcely equal to the least conversation; and if any exciting topic was touched upon, he became delirious.—The eyes were bloodshot; he could not bear light or noise; said that his nights were almost sleepless; that his brain often "seemed on fire;" and that his greatest comfort was to have his shaved head washed with the coldest water. He felt his chest bound as if with cords; the breathing was uneasy; cough was frequent and exhausting; the expectoration was in very large quantity, of highly puriform appearance, much covered with blood, and of offensive odor. The pulse was from 120 to 130; the animal heat 102 degrees.—Hectic fever was urgent, and on most nights the perspiration was excessive. The urine was of a dark color, and deposited lateritious sediment in the greatest abundance. On the right side, over the upper part, the sound was dull; the voice gave much resonance to the stethoscope near the axilla. It seemed very probable that some ulceration had taken place. He was not at this time equal to the

---

*Since writing the above, I have seen this gentleman, and find him quite well. His pulse is 68.

task of inhaling. I directed a blister to the chest, and the following mixture:—

℞. Potassæ Bicarbon. g. c. viii.; Succ. Limon, ℥ij.; Mist. Amygd. ℥iv.; Syrupi Tolutan. ʒij.; Acid. Hydrocyan, *m* x; Gutt. Nigr. gtt. xv.; Potassæ Nitrat. ℈ij. M.

Of this two tablespoonfulls were taken every four hours. He derived great relief from this medicine; but his sleep being still deficient, I directed the use of the morphine syrup at night, and its effects were most satisfactory.

He gradually improved in general health; but as the brain acquired a more healthy condition, the pulmonary symptoms became more urgent. The cough sometimes continued for an hour without ceasing; and the expectoration, which was uniformly more or less colored, was in quantity, upwards of a pint in each 24 hours.

I was resolved not to delay longer the trial of inhalation, and began with a small proportion of iodine, joined with my usual preparation of conium, the saturated tincture. At first he experienced great giddiness and sickness, and could only inhale for five minutes. He was in so weak and nervous a state (hardly able to raise himself in bed) that he was timid, and alarmed at the idea of the new treatment.—With better courage, however, he resumed it on the following day; and I was highly gratified to hear him, in a short time, express in glowing terms the delightful relief which he experienced from inhaling, which he said not only relieved his cough and breathing, but "calmed him all over." Many of the symptoms remained urgent for a week;—the quick pulse; the breathing easily hurried; the cough much excited by continued conversation; hectic fever at mid-day severe; perspiration at night excessive. But some appetite returned. There was more tranquility of the nervous system, and much sleep was procured at night. Some decoction of bark had been added to the mixture. The bowels required regulation; and a pill with *pilul. aloes c. myrrh.* and *pulv. jacob.* answered perfectly. In other cases I have mentioned the remarkable reduction in quantity, and alteration in quality, speedily produced in the sputum by the influence of the iodine inhalation; but I never witnessed this effect more strikingly produced than in the present instance. Within three days the quantity was lessened by one-half, and it was much less colored. At the end of a week it did not amount to more than four ounces, and in another ten days it was reduced to an ounce, with here and there only streaks of blood.

I am happy to add, that the patient is advanced towards convalescence, and, I hope, may, with great care on his own part, he restored to health. He has gained flesh and strength, and has a good appetite. When he is in a state of perfect quietude the pulse is below 80, but is soon quickened by a little exertion. The animal heat is now only 98 degrees. He is in good spirits, and is confident of recovery, but he still has cough, with sometimes, colored expectoration; he has now and then copious night sweats; and after sitting up some hours his ankles become swollen. He continues to inhale regularly, and with unabated satisfaction. He uses the lotion for the chest, and the flesh-brush, with sensible benefit. He takes sulphate of quinine with sulphuric acid, &c., in the day, and the morphine syrup at night. He is quite free from hectic fever, and pursues a highly restorative diet with evident advantage.

Case IV.—A gentleman, aged 24, of circular chest, of the mixed temperament, often wearing in his cheeks a color like hectic flush, of a very consumptive family, was attacked with troublesome cough about four years ago. The expectoration was occasionally colored with blood, and he found for the first time that his breathing became distressingly hurried by slight exertion. He had lost flesh and strength within a short period, and was much alarmed, as were also his friends, with the dread of pulmonary consumption, from which a brother and sister had died. The indications afforded by auscultation rendered it almost certain that his lungs were tuberculated, although not *en masse.* He inhaled iodine and conium with the greatest advantage; but being of an active disposition, and disliking confinement within doors, he went to Maderia, where he passed the winter and spring, two years in succession. The last winter he passed at Lisbon. During his residence at that place he caught cold, which was followed by cough, attended with colored expectoration, (which he described as a spitting of blood,) every day for a month. He sent to London for an inhaler, the mixture of iodine, tincture of conium, and the internal medicines which I had prescribed for him on a former occasion.

He did not receive the articles till the expiration of a month.—A medical friend in attendance upon him used his strongest persuasions to dissuade him from inhaling, under the circumstances of a troublesome cough, attended with colored expectoration, assuring him that in all probability a dangerous hæmorrhage from the lungs would follow. He, however, fortified by his former experience, was resolved to adopt the treatment; and, accordingly, he inhaled the solution of iodine, with the addition of tincture of conium. He informs me that after the space of three days, the blood entirely disappeared; the cough was relieved, and in a short time he recovered his health.

Case V.—A young woman of delicate frame, and rather narrow chest, in the year 1825, suffered from cough and difficulty of breathing, for which she was bled twice from the arm, and blistered repeatedly.

In 1826 blisters were used; and she took digitalis with some advantage to the shortness of her breath, but with injury to her stomach and nerves.

During the two following winters blisters were applied.

In 1830 I was consulted, when she was suffering from very troublesome cough, short breathing, a sense of tightness and of soreness in the chest, without any fever. Her breathing was like that of an asthmatic person, and always became distressed under the least exposure to a foggy atmosphere. She was getting thin and weak, and she had also a consumptive look.

I directed the inhalation of iodine and conium, in conjunction with the washing and friction of the chest; and no other treatment was employed. Suffice it to say, that she recovered in the most favorable manner.

Capt. Kater, in whose service the patient lived, informs me that since the period of my attendance she has but rarely been affected with her complaint; and has, on each occasion, obtained relief in a few days from having recourse to the inhalation.

I could multiply examples equally in favor of the value of inhalation of iodine and conium, as those which I have stated; but I have no right to trespass further on your pages; and I trust that I have offered sufficient proof to satisfy every candid reader.

I will take the opportunity of mentioning that the patients whose cases of confirmed tubercular phthisis, with ulcerated cavities, are detailed in the additional part of my second edition, at p. 138, c. i; p. 184, c. ix; p. 194, c. xi; all continue to enjoy their recovered health, bearing the most happy testimony* to the benefits derived by them from inhalation and the collateral treatment.

It is my earnest recommendation to the profession to give a fair trial to the practice, and to use due *perseverance*, to which principle of acting I owe so much of my success. I am certain that inhalation is often abandoned too hastily, and for very insufficient reasons.

Nor should any one allow himself to think slightly of the methcd of treatment because in many cases it will not succeed. No human means can avail in the worst cases of pulmonary consumption; but it is my sincere conviction that the best chance would be afforded to the patient by the adoption of the particular and general plan which I have laid down; and, certainly, it will be found to render the comfort of largely mitigating the symptoms, even when the force of disease is too aggravated to admit of cure.—*Lon. Lancet.—Bos. Med. and Sur. Journal.*

*Wimpole Street, Dec.* 6, 1834.

15. *Mercurial Inunction in Erysipelas.* By Adolphus Taylor, M. R. C. S. L.—The pages of the Lancet for the year 1832, No. 463, afforded me an opportunity of advocating the employment of the mercurial ointment in erysipelas. I am happy to observe that the attention of the profession has, since that period, directed itself with success to the adoption of a remedy which has proved itself as useful as it is simple in its nature: and it is with much satisfaction I perceive that your very valuable journal has been instrumental in procuring its adoption by eminent practitioners in the sister countries.

The opportunities afforded me of witnessing the disease in question, since the above mentioned year, have not been few; and the facts they

---

*The several patients who have recovered have expressed their desire that I should refer to them any consumptive invalids, or their friends, who might wish to be assured upon the advantages of the inhaling treatment.

have exhibited may not be uninteresting to the medical world; or, at all events, may lead some of its members, who are favored with ample opportunities of encountering this troublesome malady, to investigate the circumstances more closely than other engagements have allowed me to do. A statement of individual cases would trespass too much upon your columns, to the exclusion of matter equally important. To avoid such injustice, I will therefore, concentrate in a brief account those guiding principles which have fallen under my observation.

In a simple case of *idiopathic* erysipelas, when seen before the formation of vesicles, the constitution not being impaired by excesses of any kind, I have seldom found it necessary to employ any other remedy than the *ung. hydr. fort.*, paying attention to the state of the bowels and enjoining low diet. I cannot recollect ever being necessitated to make use of more than five applications, and frequently not more than three were required; in these cases I did not find that the mercury at all affected the mouth. When, however, the patient (as is but too often the case in erysipelas) had weakened his constitution by intemperance, and there was much disposition to œdematous effusion; or when any other cause had abstracted a portion of that power which the body possesses of resisting disease; *then*, where stimuli were indicated, a perseverance in the use of the same remedy has indeed shown its most decided advantages, exhibiting a striking influence over the progress of the disease, and preventing its extending to internal and more important structures. In such cases, I have almost invariably found the mouth become affected, while the decline of the symptom has, in a marked manner, followed the appearance of ptyalism. From mercury in no other form have I met with the success which has resulted from the application of *ung. hydr. fort.* The other preparations of the same metal were either discontinued from their too stimulating nature, or have seemed to be ineffectual in arresting the progress of the malady. Perhaps, however, I might suggest that were the case to have proceeded to such extent as to show a tendency to gangrene, the *linimentum hydrargyri* of the Pharmacopœia would, by its combination of mercury and camphor, be a preparation advantageously adapted to the case.

Unless the ointment be applied *immediately to the part affected*, I have not observed any beneficial results from its employment, and in some cases where, for experiment sake, I ordered friction with the mercurial ointment to the groin, for erysipelas on the leg, it was not only of no service in arresting the disease, but the latter went on increasing under the treatment. As a rule, with respect to the *temperature* practised towards the affected part, it will be well to mention that the ordinary atmospheric standard has invariably been better suited to the cases that have occurred within my view. About a month since, I had an opportunity of observing, in a very pointed manner, the good effect of this remedy. It was in the case of an ill-nourished, attenuated, pauper female, who had been a frequent subject of erysipelas: in this case, it made its appearance on the right thigh, and was rapidly extending. I had the satisfaction of arresting its progress by the mercurial ointment alone, for no other remedies were employed but a cretaceous mixture, to remove an accompanying diarrhœa: under different plans of treatment she acknowledged that her illnesses lasted, and with far greater severity, for a much longer time than the period (a few days) she remained under my care.

Such are the gleanings I have to offer to the notice of my fellow brethren, and to their judicious conduct I leave the power which experience has taught me the mercurial ointment possesses to alleviate suffering, and palliate one of the most inveterate diseases to which humanity is liable.—*Lon. Lancet.—Bos. Med. and Sur. Journal.*

16. *Fatal Effusion of Blood into the Pericardium.* By Dr. Carson, of Liverpool.—The following very interesting case we copy from the first number of the Liverpool Medical Journal—a new contemporary which augurs well, if we may judge by the first specimen.

"Mr. W. a gentleman about 52 years of age, of a tall and robust form, clear complexion, subject occasionally to dyspeptic affections; though of very regular and temperate habits; of an active disposition, though his occupation was sedentary and confining; had been for 12 months affected with considerable anxiety of mind, in consequence of the doubtful issue of some building speculations. Towards the end of Lent, which he had rigidly observed according to the injunctions of the Catholic church, on the 11th of March, a day exempted from the prohibitions respecting diet, he had eaten freely of beef-steaks, with onion sauce. He was at that meal sparing as usual in the use of wine. On the evening of the following day, he was engaged in a fatiguing and rather anxious way, with the business of a club, of which he was treasurer. On his return from the club, about 11 o'clock at night, in company with two of his friends, when he had nearly reached his own house, he was seized with faintness and debility to such a degree, that without the assistance of the friends who accompanied him, he would not have been able to have kept his feet. Soon after his arrival at his house, he was visited by Mr. Bromilow, his medical attendant. He described himself as faint and exhausted; complained of an obtuse, heavy pain at the precordia, and was affected with flatulent eructations. His respiration was free, his pulse 70, and regular, though weak. He had no affection of the head, nor pain any where, excepting as described in the chest. His bowels had been opened that day. Mr. Bromilow ordered an antispasmodic draught; and left him with directions to take something warm, and go to bed. He took the draught and a weak glass of brandy and water. At 3 o'clock he sent for Mr. B. again, and, as the pain in the chest was not abated, he expressed a wish to be bled, which Mr. B. agreed to, more with the hope of satisfying his mind than from any great necessity for that measure being indicated by the symptoms. He lost a pint of blood. An opiate was then administered. At this visit Mr. B examined the chest more minutely. He applied his ear to the different regions of the naked chest, but perceiving no unusual sound, or vibrations, concluded that the heart, lungs, and large vessels were in a sound state. At 5 o'clock

A. M. I visited him. He felt cold, perspired gently, and chiefly complained of a pain in the chest, which he described as wearisome and oppressive. It was not increased by taking a full inspiration. He had vomited a little in the course of the night, and had discharged some of the onion sauce he had taken the day preceding the attack. He was much troubled with flatulency, and belched frequently, but was not relieved by it so far as regarded the pain in the chest. His pulse was regular; the heat of the body natural; and respiration good. He had had no sleep.

From the information given by Mr. Bromilow, connected with my own observation, I considered that nothing could be indicated by the symptoms beyond an affection of the stomach, which is known to exhibit itself in such anomalous forms. He took four grains of calomel, and two of opium. We visited him again at half after eleven o'clock. He had had little sleep. The symptoms remained the same. He was ordered an aperient mixture, and we proposed to visit him again at seven o'clock. At this visit, I replied to the anxious inquiries of the family—that we did not see any cause for alarm: that the complaint seemed to arise from indigestion; and that I had no doubt he would recover. At three o'clock in the afternoon he sent for Mr. Bromilow, as the pain still continued unabated, and wished to know if he might have any thing to rub the part with. The bowels had not been opened, and he had had little or no sleep. A short time before seven o'clock, the hour at which we had proposed to visit him, and at which I was prevented from attendance by an urgent call to a distant part of the country, Mr. W. was seized with what the family conceived to be a fit: and a short time after the arrival of Mr. Bromilow, expired. In consequence of my unavoidable absence, other physicians were called in, and two arrived, but not until after the death of the patient. I applied for permission to open the body, which was granted. The body was examined twenty-four hours after death, by Mr. Bromilow, in my presence, and in that of my son, Dr. Carson, jun. The following were the appearances on dissection.

Upon opening the chest, the lungs on both sides were perfectly sound and collapsed. But, notwithstanding the collapse, the chest was filled more than it usually is when the lungs are sound. This indicated the existence of some foreign substance or morbid enlargement of some of the organs. The pericardium was found accordingly to be immensely distended by some fluid, which, when this bag was opened, was found to be blood, partly liquid and partly coagulated: the quantity was not less than three pints. It was purely blood, without the admixture of any fluid indicating inflammatory action. The external surface of the heart, and internal surface of the pericardium, were examined carefully, but no ruptured vessels, from which the blood might have flowed, were discoverable on either of these surfaces. The heart itself was perfectly sound, the valves were in good condition, and no disease existed in any of the large vessels. The lungs were free from adhesions, and were every where sound. The other viscera were in a sound state. A great deal of care and time were expended in trying to discover the source from which the blood had flowed into the pericardium, but in vain: a slight ecchymosis was observed about the root of the pulmonary artery. Dr. Baillie, in his *Morbid Anatomy*, says, 'Cases have occurred, though very rarely, in which a large quantity of blood has been accumulated in the cavity of the pericardium, but where no rupture could be discovered after the most diligent search, either in the heart itself, or in any of its vessels. This appears very wonderful, and not at all what any person would expect *a priori.* Two conjectures have occurred to me, to explain this phenomenon: 1st, that the blood vessels on the surface of the heart have lost their compactness of tissue, so that the blood may have escaped by transudation. The other is, that the blood may have been poured out by the extremities of the small vessels, opening on the surface of that part chiefly of the pericardium forming the immediate cover of the heart, from their orifices having been to a very uncommon degree relaxed.'

There is a case related by Dr. Alston, in the sixth volume of the Edinburgh Medical Essays, in which the disease of the chest was of long standing. Three pints of blood, which was partly coagulated and partly mixed with lymph, were found in the pericardium. No ruptured vessel was discovered either on the outer surface of the heart, or the inner surface of the pericardium. Upon pressing the heart, a bloody serum oozed out of a great many orifices on its surface, and principally near its base. No disease was discovered in the interior of the heart or large vessels. Dr. Baillie refers to two cases of extravasation of blood into the cavity of the pericardium, in which the source of the hæmorrhage could not, after the most careful examination, be discovered. In both these, functional disease of the heart had been observed for some time previous to the death of the patient. Vide Medical observer, vol. 10, p. 330. Memoirs of Medical Society, vol. 1, p. 238.

Various opinions have been advanced respecting the sources from which, in the above cases, the blood was derived. One of the suppositions made by Dr. Baillie appears to me to approach the nearest to the truth, which is that the blood had oozed out of the small vessels on the internal surface of the pericardium immediately covering the heart. It is probable, I think, that the oozing, particularly in the case now narrated, arose from the condition of the blood, and the relaxed state of the fibres. It would appear that the disease was general, and that the shivering, faintness, and depression of spirits, were not the effects of the flow of blood into the pericardium, but that this last was, like the affections stated, the effect or symptom of the general disease,—that in fact there existed a morbid state of the whole systém, similar to that which takes place in purpura, in some kinds of epistaxis, hæmatemesis, and in bleeding from the bowels in typhus fever. The pain in the chest was in the first place, occasioned by the admission of blood into a cavity not accustomed to the stimulus of that fluid. There is no reason to suppose that the action of the heart would be mechanically affected until the quantity of the blood was pretty considerable; for the blood would readily follow the dilatation of the pericardium, occasioned by the elasticity of the lungs, when the chambers of the heart had finished their contractions. No sound was perceived, on carefully examining the chest. Indeed no sound could be excited, as no fluid was poured from one vessel into another. For as the auricles expand as the ventricles contract, the change of place in the constituents of the fluid in the pericardium would be inconsiderable, and made with quietness.

There does not appear to be any symptom in this case that would have warranted the medical attendants in giving an unfavorable prognosis. As a matter of prudence, a less favorable one might have been made, but the same prudence would not permit the expression of a favorable prognosis in any case whatever."

Upon this case we may be permitted to make a remark or two. We have no fault whatever to find with the treatment of the case, nor do we differ from the highly talented narrator of it as to its pathology—or rather etiology. But we must say that the exploration of the chest was incomplete, and that *percussion*, added to auscultation, would *undoubtedly* have led to a grave prognosis. The state of the pericardium would have caused a dull sound, on *percussion*, over a very unusual space, and would have caused the greatest alarm in the mind of the medical practitioner accustomed to thoracic exploration. The want of any symptoms of enlargement of the heart, too, would have led to the inevitable conclusion that there was fluid of some kind in the pericardium. Thus, then, we see the great value of auscultation and percussion. In the present case, it would have saved the medical attendants from the suspicion (and indeed a justly founded one,) that they were unaware of the danger, and consequently of the nature of the disease. Nothing could have saved the life of the patient, had the physician been perfectly acquainted with the true state of the case; but his diagnostic and prognostic skill would not have suffered, had an accurate exploration been made.

We may take this opportunity of saying that, were we to be deprived of one of the two means of exploration, we would far rather give up auscultation than percussion. The latter is far more valuable than the former, though they prove auxiliary to each other.

We are sure that Dr. Carson will excuse these remarks, as they can have no other object than the good of the profession. Dr. C.'s reputation is far above the reach of censure, were we inclined to be censorious, which we are not. His brethren are deeply indebted to him for the candid manner in which he has narrated this interesting case.—*Med. Chir. Rev.*

17. *Ulcers of the Leg.*—From a little volume of considerable practical utility, entitled "Observations on the Ulcerative Process and its Treatment, particularly when affecting the Leg," by Mr. Eccles, we make the following extract, as a sample of the matter and manner of this unostentatious publication.

"The cases in which the morbid condition of the condensed cellular tissue forming the cup of the ulcer appears to be the cause of continuance of the disease, are distinguished by the induration of the edges, the scantiness of the secreted matter, and the insensibility of the sore. There can be little doubt that bandaging and various stimuli have some effect on the cellular structure in question, as well as on the sanguineous and secernent vessels; but there are two agents which I find of particular efficacy in removing the more obstinate peculiarities of such cases. These are mercury and iodine. The former is well known to possess the power of lessening induration when given internally, and some have thought that the same efficacy is attached to its local application. After much experience, I am, however, brought to the conclusion that mercurial dressings are void of any specific utility analagous to the constitutional action of the remedy; that they stimulate in peculiar modes, but perform no important part in effecting the removal of indurated cellular tissue. The local effect of mercury is extremely great in changing the character of sores in general, and in the form I am describing, it is very salutary. We shall find, however, that if its action be not immediate, no good purpose will be answered by its continued employment.

Iodine, internally administered or externally applied, and even in those forms which have lately been described as inefficacious, I have always found of great service in removing this species of induration. It often requires to be used for a tedious length of time, to be remitted should it produce much constitutional derangement, and again employed when the state of the general health will permit. I have not met with a case in which iodine has failed to remove the induration; but if such were to occur, it would perhaps be advisable to remove the indurated structure with the knife. This has been done, and certainly with perfect success, the new wound healing rapidly. But so decisive a practice would only be justifiable where every other method had repeatedly failed."

There are many judicious observations in this book on the ulcerative process, as it affects the different animal structures, which deserve the attention of the young surgeon.—*Med. Chir. Rev.*

18. *Method of Reducing Dislocations of the Head of the Humerus, practised at the Bristol Infirmary.*—This is detailed by Mr. W. F. Morgan, late house-surgeon to the institution.

"As soon as the patient is seen, and the nature of his injury ascertained, he is placed sideways on a common chair, with the dislocated arm hanging over its back, which is padded for the reception and support of the axilla. A reel-towel is then fastened on the arm, immediately above the condyles, by a running noose, with the knot outwards; and its lower part is formed into a loop some way above the ground, to receive the foot of the surgeon. One or two assistants press on the shoulder, much in the same way as Mr. Toogood advises, to fix the scapula and prevent the patient from rising, and the surgeon puts his foot, or, occasionally, his knee, into the loop of the towel, and gradually and unremittingly bears on it with the whole weight of his body.

In a very little time, generally within three or four minutes, the head of the bone slips into its place with an audible jerk; the patient's sufferings are moderate, and of short duration, and the use of his arm is soon restored.

In dislocations of long standing, of course other and more powerful means are requisite; but, in several of the cases to which I have alluded, many hours, and even days had elapsed, before the patients applied for relief. I have, likewise, notes of the case of a strong and muscular man, who was admitted with a dislocation of the shoulder, complicated with fracture of the humerus at its middle, in whom the reduction was effected as readily as usual, by binding up the arm with splints, and applying the towel on the outside of them."

In the great majority of recent cases, the reduction is readily, simply, and quietly effected by the heel of the surgeon in the axilla. No towels are necessary—no further preparation is required than removal of the surgeon's boot—no other requisite than a moderately-clean pair of stockings.

We now take leave of this volume of Transactions for the present. There are many articles for notice—some for criticism, in our next. Till that makes its appearance we wish the contributors adieu. It would appear ill-natured to hint a defect on parting, yet censorious readers might complain of prolixity in several of the articles.

19. *On the Certainty and Safety with which the Operation for the Extraction of a Cataract from the Human Eye may be performed, and on the Means by which it is to be accomplished. By G. J. Guthrie, F. R. S. &c.*—This pamphlet is printed for the benefit of the Ophthalmic Hospital, a valuable institution, much wanted and much prized at the western end of the metropolis, and which owes its maturity, if not its birth, to Mr. Guthrie. We have so frequently introduced this able surgeon to the notice of our readers, that we may proceed, without farther preface, to extract from the pages of the work before us a few of the practical recommendations contained in it.

Mr. Guthrie alludes to Wenzell's remark, that an oculist must spoil a hatful of eyes before he can extract a cataract well. This Mr. G. believes must be the case, if the surgeon implicitly pursues the directions which were given at that period. We can well believe that Mr. Guthrie's hatful is a small one—a low-crowned modern beaver, not admitting of comparison with the huge and capacious structures which formerly surmounted the human head.

We shall endeavor to present Mr. Guthrie's sentiments on some of the points which have excited most discussion amongst oculists, as well as upon those in which his experience may render his opinion valuable.

The following are his directions respecting the manner in which the patient should be prepared for the operation.

"It appears to me, that the same preparation only is required that would be considered necessary previously to the performance of any surgical operation of importance, and particularly about the head. If the patient is in good health, and free from any appearance of disease, nothing more is wanting than a couple of gentle doses of physic, and moderate abstinence during the week preceding the operation. If the patient has lived regularly well, and is disposed to be of a full habit, twelve ounces of blood may be taken from the arm the evening before the operation. If the health is impaired from an irregular mode of life, it should be amended. If a fit of the gout is impending, or is supposed likely to occur, it should be allowed to pass over before the operation is performed; or if the patient has a strong determination of blood to the head, he should be cupped a few days before it is done, in addition to the bleeding recommended on the evening preceding the operation; and the same or greater precautions should be taken when there are indications of a tendency towards paralysis or apoplexy. These are, however, only the ordinary precautions which a well-informed surgeon would take in all other cases. When the operation is to be done on nervous persons, care must be taken on the other hand to prevent any undue depression of strength or spirits."

Mr. Guthrie was formerly of opinion that both eyes should not be operated on at once. He was not so certain of success then as now, and he does recommend the simultaneous performance of the operations, chiefly for the following reasons:—If an accident should occur, and a very vigorous or prolonged after-treatment be necessary, the patient, whatever may be the result, will not readily submit to a second: and, if an elderly person, will not be in a state to submit for a great length of time afterwards; and if that second operation should be unlucky, requiring also a vigorous after-treatment, the loss of health, and ultimately that of life, may be the consequence. Elderly persons can generally bear a vigorous treatment *once* well, and recover from it completely; but they cannot often bear and recover from it *twice*.

The operation of extraction should always be performed with the corneal incision in the direction upwards. Mr. Guthrie's directions for the management of the lids and of the eye are most judicious.

"For the operation on the right eye, the surgeon should place himself behind the patient, and he will usually find it necessary to stand on a stool, in order to raise himself to such a height, that he may readily lean over, and have his hands at perfect ease; and in that position and distance from his own head or chest, which is most convenient to him. The patient's head being a little inclined backwards, and duly, although gently and comfortably supported by the cushion, or back of the chair; the surgeon leaning over from behind, brings the *two* fore-fingers of the left hand over the forehead gently down on the eyelid, and raises it up slowly and tenderly, so as to fix it ultimately against the upper edge of the orbit; and to be able to retain it there so perfectly with the end of *the* fore-finger only, that the patient cannot lower it or close the eyelid by any effort he can make. He should also be able to do this and to make a little pressure on the eyeball, in order to fix it at the moment the incision is begun. As soon as the index finger is in this position, the second finger leaves the upper, and lowers the under lid, and fixes it against the edge of the orbit below. The eye is thus completely exposed, and may be almost fixed between the two fingers. To do all this well, requires a certain degree of practice, but which is very easily acquired. It must be done very gently, very tenderly, and without giving pain, or almost uneasiness. The error usually committed, is in using too much force with the extremity of the finger, which gives pain, and makes the patient swerve; and it is an error of such great importance, that the surgeon must practise this part of the operation until he feels that he does it as a matter of art, not of force.

The left eye may be opened, and fixed in a similar manner; or the surgeon standing before the patient, raises the upper lid with the side of the fore-finger of the left hand, and depresses the under lid with the thumb, the hand being over the nose. The pressure of the fore-finger tends to fix the eye at the same time, and to render it as immoveable as possible, and this mode of proceeding I generally adopt in preference, for the left eye."

Mr. Guthrie ably exposes the fallacy of the popular belief, that the eye is a very delicate and a very sensitive organ, and hints that the notion has been propagated and fostered for interested purposes. He observes that such a notion cannot long be entertained, by any who are in the habit of witnessing the performance of operations on the organ. Mr. G. observes, that full exposure of the eye diminishes, in a great degree, its sensibility. He recommends the surgeon to acquire the power of operating, without the assistance of another in steadying the eye; unity of purpose is gained by this. The knife which he prefers is spear-pointed, and blunt on the back.

"The width depends on the maker, my rule being that it shall be as broad as the perfection of the point will admit, and the artist finds that he cannot put the best point on a broad instrument, so that it is essentially rather a narrow knife. It should be of that degree of equal thickness which will admit of its passing through the cornea with the greatest facility, and of its bearing the very best possible point and edge. Every thing in this operation depends on the point of the knife, for the operation must fail either partially or totally, if the point of the knife is not a good one; nothing else signifies so materially, and it must be attended to. The surgeon should never depend on the instrument maker: he should try it on the fine piece of thin leather which ought to have a place in every case of eye instruments. Through this it should pass as through water, without the slightest sound; for if the slightest sound be heard, the knife is unfit for use. Held between the fore-finger and thumb, with the little finger resting outside the orbit, the surgeon awaits calmly the steadying of the eye, which should be fixed nearly in the centre, the pupil looking directly forwards. The flat part of the cornea-knife may now be made to touch the face of the cornea as the probe did before, in order to ascertain that the patient is totally free from alarm, and that the eye is steady."

Mr. G. sides with the advocates for the use of belladonna before the operation. It should be applied on the previous evening. After stating that the upper half of the cornea must be divided, Mr. Guthrie makes some lively remarks on the mode in which the knife is to be made to enter it.

"The manner of entering the point of the knife is disputed. The cornea being composed of several layers, constituting a substance of a certain thickness, there is some danger of passing the knife between the layers, and not across the anterior chamber of the eye in front of the iris, if care be not taken that the cornea is fairly penetrated in the very first entering of the knife; which accident may happen, if the anterior chamber is small, and the iris is close to the cornea. In order to avoid this error, modern authors (I believe, without an exception,) recommend that the knife should be entered in the direction of the iris, as if the point were to be carried directly against it, but that as soon as the cornea is penetrated, and the point is in the anterior chamber, the handle of the knife is to undergo a sort of almost imperceptible depression towards the temple, by means of which, the blade is to be placed with its flat surfaces parallel to the iris, across the anterior chamber; and it is said that the more quickly this is done, the less chance is there of the escape of the aqueous humor. To all this I object in the most decided manner; the gentlemen who think they do as they say, are, I believe, mistaken; and when they do, they do nothing but mischief. The very turning, or attempting to turn the knife, generally leads to the escape of the aqueous humor, the very thing they want to preserve, and to all the evils it is most desirable to avoid. The knife should in my opinion, be held flat, and the point is to be introduced steadily in the same direction at the proper place; and if the operator can neither see nor feel when it has penetrated the cornea, and is in the anterior chamber, the sooner he abandons operating the better. If a man has eyes, he can see when the point of the knife has entered the anterior chamber; and if he has fingers he will feel when the resistance offered by the cornea is overcome. The young surgeon, instead of practising these manœuvres, which are worse than useless, should practice on sheep's eyes, until he has acquired that tact, which will enable him to know when his knife is in the right place."

Mr. Guthrie alludes, in a forcible manner, to the disposition in the eye to turn inwards at the moment of the incision, and enjoins the utmost coolness on the part of the operator. He dwells very strongly on the fact that the inner layer of the cornea is more tough than the outer, and gives many interesting practical hints to the operator. We regret that we cannot introduce them. For the following piece of information and advice, we must certainly make room.

"When the perforation or penetration is completed, and well done, the patient and surgeon are in a very different relative situation to each other; before it was done, the eye was almost entirely under the control of the patient; after it is done, the eye is almost entirely under the control of the surgeon. It moves with, and of course follows the motions of the knife, and if it has turned inwards, even into the very internal angle, the knife can bring it gently back to its due central position, but not without the loss of the whole of the aqueous humor; and the iris is now seen bulging forwards on the edge of the knife, sometimes even overlapping it. It is at this point that a young operator is confounded; he sees that the iris must be cut, if it cannot be moved out of the way; he thinks he has failed, becomes confused, hesitates, and does mischief, or withdraws the knife and abandons his operation: or if he is a clear headed person, he bethinks him of the directions he has read upon this subject, and proceeds accordingly; but he does not succeed one whit the better, and in despair, accuses himself of awkwardness. But he is not, however, the person in error —it is those who wrote the directions. I do not mean to say that these gentlemen have willingly deceived the public, but I do say it is my opinion, that they have told only half the truth. It may be that they did not know the other half, and I have no objection to its being so considered, except that I shall be set down for a discoverer of it, whether I will or not, which I am very desirous to avoid. The directions given in such a case by the Baron de Wenzel, and which the late Mr. Ware said, were the most important in his whole book, are, that the cornea must be gently rubbed with the point of the fore-finger, which causes a contraction of the pupil and consequent drawing back of the iris, when the surgeon must complete the operation; but if the iris again fall forward, before this is accomplished, he must keep the finger on the cornea, until it is effected, by which, all danger of the iris suddenly protruding will be avoided. It is also recommended that the surgeon should wait a little, and allow the spasm to subside; and he may wait and rub, and wait again, and then rub again if he pleases, but he will rarely succeed, unless he does something more, and that is, to raise the eye, or in other words, to draw it as it were out from the orbit, whilst at the same time, he presses the cornea flat against the blade of the knife. This is the other and the best half of the secret, and without he does which, he will not succeed in disentangling the iris. A little consideration will, I think, show why it can only be done in this manner. When the aqueous humor has escaped, the cornea becomes flaccid, and the remaining humors of the eye advance, or are brought forward by the action of the recti muscles, so as to press the iris against it. If the knife is between the iris and the cornea, it keeps these parts asunder, as far as its width extends, but not farther, and as it raises the cornea in every part, it would make a vacuum below its edge, between the iris and the cornea, if the iris did not rise up to fill the space, or if the air did not rush in to do it. This effect of the air, is however counteracted by the muscles acting on the eye with greater power; and the consequence is, that the iris is forced upwards into the vacant space, and the air, if any has entered, is expelled. The iris can, however, be only elevated or protruded to a certain extent, in consequence of its circular attachment, and of its disposition to contract towards its pupillary edge, or centre. When the cornea is well raised, so as in some degree to raise the eye along with it, whilst, at the same time, the cornea is pressed against the blade, the iris slides from between them, and the operation may be completed, so as to give rise to a highly successful operation. There is now another secret to be disclosed, of yet greater importance to the young operator; it is, that the effect of an injury to the iris is very greatly overrated, and that if the operation cannot be completed without injuring it, the injury must be committed. The real truth is, that *no good operators* in this great city of London do otherwise. When the iris bulges much over the edge of the knife, it is often not possible to get it quite clear, by any effort, exertion, or dexterity, on the part of the operator. If any man says otherwise, I do not hesitate to say that he is in error, and that his own operations, if he has ever done any, will show it. This being the case, the operator has only a choice of evils, to proceed under any circumstances, or to abandon the operation. This I have done, and the consequence was, that as much inflammation followed as if the operation had been duly performed; and the eye was not upon the whole in a very favorable state for a future operation, the iris being usually a little injured, and the consequent inflammation rendering the pupil less regular and dilatable than it ought to be. If the operation is on the contrary completed, the iris is slightly shaved as the knife advances, or a piece may even be cut out, but the patient will, nevertheless, have a very good eye. The cut in the iris will often not be discernible, unless the upper lid is raised to look for it, and he will see remarkably well, after a very speedy recovery. In fine, I may say the operation is always done in this manner by all those who know what they are about, and the eyes of persons of all ranks who have been operated upon, either in London or on the continent, prove it. It is possible that the operators may not know what they have done, or how they did it; they may have supposed they were doing one thing, while they were actually doing another. I do not dispute this, or anything else, in the least, but I write to give such information to young surgeons as will enable them to operate successfully, and to know why and wherefore they are successful."

For many valuable observations, and for some particulars respecting a patient on whom De Graefe operated at Mr. Guthrie's request, whilst Mr. G. himself extracted the lens of another, we must refer the curious reader to the pamphlet itself.

There is a circumstance attending the protrusion of the vitreous humor, with which young operators should be familiar.

"The lens," says our author, "does not however always come out so easily and regularly. It sometimes happens that instead of rising up by its upper edge, the vitreous humor, which appears black because it is transparent, and allows the black pigment beneath to be seen through, is perceived pushing forwards between it and the iris.—Nothing can prevent a portion of this vitreous humor being protruded or expelled, and no attention need be paid in order to obviate it, for it cannot be done. But attention must be paid to the fact, that its expulsion under pressure will not in general be accompanied by that of the lens, which, having lost its support, will sink down towards the bottom of the eye, and must infallibly cause its destruction by inflammation if not removed. The surgeon aware of this circumstance, and knowing that pressure of any kind, that even the mere action of the muscles, will cause the expulsion of the vitreous humor without the lens, passes either the large or the small hook under the edge of the lens, through the vitreous humor, which begins to escape when its enclosing membrane is pierced by the hook, the point of which is to be fairly stuck into the under part of the lens, and thus drawn out. A portion of vitreous humor must of course escape, it cannot be prevented, it was inevitable from the first; but the great object, the extraction of the lens, has been attained. If the surgeon hesitates, and does not calmly and steadily introduce his instrument, and hook the lens at once, the vitreous humor begins to escape, the lens sinks, and the eye will be lost, if he does not instantly pass the hook through the pupil, and hook the lens, as he would catch a fish with a landing hook.—There is no alternative, it must be done, or the eye will be destroyed."

Mr. Guthrie observes, that the loss of a portion of the vitreous humor has been a surgical bugbear in the operation. It is an evil, but not a very great one. The principal inconvenience accruing from it is an irregularity of the pupil, which does not in many instances impair vision. If a very small portion of vitreous humor be lost, it is usually followed by severe inflammation; if a large quantity has escaped, the inflammation is usually less. The pupil is apt to be drawn in the direction in which the expulsion or evacuation has occurred, and usually retains more or less of that distortion. The best method of causing its return to its natural place, is to allow the lid to fall, and then to rub it very gently with a soft and wet sponge, for two or three minutes, which often brings it very nearly back to its central position. Another and a greater inconvenience arising from the evacuation of the vitreous humor, is, that a portion interposes between the cut edges of the cornea when they are supposed to be placed in apposition; and whilst it prevents a rapid union between these parts, it also tends, by allowing the newly secreted aqueous humor to escape, to draw the iris into the line of the incision. If this takes place, the iris adheres to the cut surface, the pupil is permanently and more completely drawn in that direction, and the anterior chamber of the aqueous humor is less perfect, both in shape and appearance, in consequence of the cornea and the iris being brought nearer to each other. This inconvenience arising from the interposition of the vitreous humor between the edges of the incision in the cornea, is to be avoided as far as it can be done, by carefully removing with the curette any part of it which can be seen, and by gently rubbing the eye in the way directed, in order to cause the pupil to return to its proper place.

The adjustment of the cornea is of course a matter of importance. The eye should appear flat. If it does not the improper fulness arises from some displacement of the vitreous humor, which has not burst the membrane in which it is inclosed, but has been pushed forwards by the action of the muscles of the eye, so as to cause the iris to project; the flap of the cornea is seen lying upon it, and is separated by it from the other edge, with which it ought to be in apposition. Adhesions of the iris and many other evils will ensue unless this be prevented. The vitreous humor must be pressed back into its place by gentle friction on the eyelid covering the ball, by which it may in general be accomplished; but if it will not yield to this treatment, the hook should be introduced through the pupil, and the membrane of the vitreous humor should be punctured, so as to allow the evacuation of one fifth of it, when the eye flattens, and the cut edges of the cornea can be brought into apposition. The extent of evil is now known, and can be duly estimated, and guarded against. In the former case it cannot; and of the two evils, the last should always be chosen, inasmuch as it will lead to the recovery of the patient with a good eye, which the other cannot do.

"The eyelid, being at last closed, is to be retained in that situation by compress and bandage; and it is not to be raised, or the eye opened, for a certain length of time, the duration of which is different, according to different writers. It ought to depend on circumstances; the earliest period is on the third day, but the fourth is much better; and if any difficulties have been experienced during the operation, and there is reason to suspect that union has not taken place, the lid should not be raised for at least a week; although the lower lid may be depressed, to enable the surgeon to see the state of the ball of the eye, and to prevent the accumulation of fluids in it. I never open the eye before the fourth day, although I frequently depress and clear the lower lid on the third, and obtain a view of the globe, the whiteness of which is the best sign of the absence of inflammation. When the eyelid is raised too soon, that is before the union of the cut parts of the cornea is complete, the movement of the globe of the eye, which inevitably follows the raising of the lid, often separates the incompletely united parts; pain of a more or less acute nature is soon felt in the line of the incision, inflammation follows, ulceration succeeds, and the cornea is only completely united by a slow process, attended by opacity, and generally by a drawing up of the pupil; all of which evils would have been avoided in a great measure by delaying the raising of the lid for two or three days, until the parts were more completely consolidated."

The state of the eye can be ascertained by the appearance of the upper lid, which should be carefully inspected twice daily.—Swelling of the lid is a test of inflammation of the organ. On the evening of the operation Mr. Guthrie usually directs the abstraction of from twelve to sixteen ounces of blood. It should never be carried so far as to occasion sickness. The eye may be occasionally fomented with a warm decoction of poppies, care being taken that the eyeball is not moved, so as to cause a separation of the divided parts; all remedies likely to cause sickness should be avoided during the course of the treatment, and active aperients for the first two or three days will, in all probability, be unnecessary, from their having been adopted previously to the performance of the operation. Mr. Guthrie strongly deprecates opening the eye or meddling with it.

Mr. Guthrie has seen one instance of the reproduction of the lens.

The practical nature of the observations contained in this pamphlet, and the candid manner in which Mr. Guthrie communicates the results of his experience to the profession, will more than excuse the length of this notice. Those who take an interest in opthalmic surgery, (and who, at the present day, do not?) will peruse the quotations we have made with much interest.—*Med. Chir. Rev.*

